# Salivary S100 calcium-binding protein beta (S100B) and neurofilament light (NfL) after acute exposure to repeated head impacts in collegiate water polo players

**DOI:** 10.1038/s41598-022-07241-0

**Published:** 2022-03-02

**Authors:** Derek C. Monroe, Elizabeth A. Thomas, Nicholas J. Cecchi, Douglas A. Granger, James W. Hicks, Steven L. Small

**Affiliations:** 1 Department of Kinesiology, University of North Carolina at Greensboro, Greensboro, NC 27402 USA; 2 Department of Neurology, University of California Irvine, Irvine, CA 92697-3957 USA; 3 Department of Epidemiology, University of California Irvine, Irvine, CA 92697-3957 USA; 4 Institute for Interdisciplinary Salivary Bioscience Research, University of California Irvine, Irvine, CA 92697-3957 USA; 5 Department of Ecology and Evolutionary Biology, University of California Irvine, Irvine, CA 92697-3957 USA; 6 Department of Bioengineering, Stanford University, Stanford, CA 94305 USA; 7 Department of Psychological Science, University of California Irvine, Irvine, CA 92697-3957 USA; 8 School of Nursing, Bloomberg School of Public Health, and School of Medicine, Johns Hopkins University, Baltimore, MD USA; 9 School of Behavioral and Brain Sciences, University of Texas at Dallas, 800 W Campbell Rd, Richardson, TX 75080 USA

**Keywords:** Biomarkers, Trauma

## Abstract

Blood-based biomarkers of brain injury may be useful for monitoring brain health in athletes at risk for concussions. Two putative biomarkers of sport-related concussion, neurofilament light (NfL), an axonal structural protein, and S100 calcium-binding protein beta (S100B), an astrocyte-derived protein, were measured in saliva, a biofluid which can be sampled in an athletic setting without the risks and burdens associated with blood sampled by venipuncture. Samples were collected from men’s and women’s collegiate water polo players (n = 65) before and after a competitive tournament. Head impacts were measured using sensors previously evaluated for use in water polo, and video recordings were independently reviewed for the purpose of validating impacts recorded by the sensors. Athletes sustained a total of 107 head impacts, all of which were asymptomatic (i.e., no athlete was diagnosed with a concussion or more serious). Post-tournament salivary NfL was directly associated with head impact frequency (RR = 1.151, p = 0.025) and cumulative head impact magnitude (RR = 1.008, p = 0.014), while controlling for baseline salivary NfL. Change in S100B was not associated with head impact exposure (RR < 1.001, p > 0.483). These patterns suggest that repeated head impacts may cause axonal injury, even in asymptomatic athletes.

## Introduction

Sport-related concussion is a specific form of mild traumatic brain injury (mTBI) caused by an impulsive force transmitted directly or indirectly to the head during athletic performance^1^. These insidious injuries are not reliably detected by common clinical imaging techniques or neuropsychological tests^2^, leading to their classification as ‘mild’. A growing body of evidence suggests that—even in the absence of overt symptoms—there is a dose-dependent association between repetitive head impacts sustained over one or more competitive seasons and changes in brain structure and function^3,4^. For some athletes, cumulative exposure to these impacts appears to contribute to the development of cognitive deficits many years later^5–7^. To better understand these risks, it is first necessary to understand the acute physiological effects of exposure, over hours and days, even when an athlete appears to be asymptomatic.

The transmission of impulsive forces to the head places a mechanical load on brain tissue that causes axonal injury^8,9^ and initiates a local neurometabolic cascade and metabolic crisis^10,11^. Experimental rodent models^12,13^ and human brain imaging studies^14,15^ indicate that blood–brain barrier disruption is also common immediately and persistently after mild head trauma. Increased blood–brain barrier permeability can lead to increased concentrations of CNS-derived molecules in the blood. Accordingly, several markers of central nervous system damage have been proposed as blood-based biomarkers for mTBI^16–20^.

S100 calcium-binding protein beta (S100B) is a brain-enriched member of the S-100 family of low molecular weight binding proteins that regulate intracellular calcium levels. S100B is one of the most extensively studied blood biomarkers for mTBI^21–25^. In one study of collegiate football players, plasma levels of S100B were higher after practice than before practice, particularly for players sustaining greater mechanical loading of the head^26^. Another study of high school football players monitored over five regular season games showed that acute, post-game increases in S100B were significantly associated with greater impact exposure^27^. However, serum S100B expression does not appear to increase reliably after mechanical loading of the head in asymptomatic athletes^28^. Another protein gaining recent attention as a biomarker for mild brain injuries is neurofilament light (NfL), a structural protein only found in large and myelinated axons of the central nervous system^29^. Several studies have demonstrated that plasma NfL is increased by axonal damage in neurodegenerative diseases^30^, after sport-related concussion^31,32^, and after asymptomatic head impacts^33–36^.

However, drawing blood to measure these proteins has many drawbacks, especially in athletic settings (i.e., field-side, pool-side, or in the locker room), where performing a venipuncture could be difficult or unsafe. Saliva is emerging as an alternative biofluid that can be more easily collected in diverse and/or remote environments, does not require trained personnel, and, unlike plasma, does not require immediate centrifugation prior to cold storage. A converging body of evidence supports saliva’s utility for differentiating between brain-injured and non-brain-injured samples^37–41^. For example, Cheng et al. reported multiple upregulated genes associated with Alzheimer’s disease in salivary extracellular vesicles sampled from mTBI patients during the acute and subacute phase of recovery^42^. To the authors’ knowledge, a relationship between putative salivary protein biomarkers of brain injury and repeated, asymptomatic head impacts has not been previously reported.

Our group has previously reported on the risks of head impact exposure in water polo, an intense, contact sport that carries a high risk of head, face, and neck injury, particularly at the elite level^43,44^. Specifically, we have observed that collegiate water polo players competing at the varsity and club levels sustain regular asymptomatic head impacts^45,46^, which may alter brain function in a dose-dependent manner over a competitive season^47^. However, the acute physiological effects of these impacts remain unknown. In this study, we test the primary hypothesis that, on an individual level, there is a dose–response relationship between the frequency and magnitude of head impact exposure and salivary expression of S100B and NfL in collegiate water polo players.

## Materials and methods

### Participants

Participants were student-athletes recruited from the rosters of two University water polo teams competing at the NCAA Division I level and two University teams competing at the collegiate ‘Club’ level during the 2018–2019 competitive season. Any active member of a water polo roster was eligible for inclusion in this prospective observational study and there were no exclusionary criteria. Participants were recruited at an athletic team meeting attended by members of the research team. Participants reported medical history, lifetime and 12-month experience in water polo, and demographic information using standard forms. All study procedures were approved by the Institutional Review Board of the University of California, Irvine and conducted according to the principles of the Declaration of Helsinki. Written informed consent was obtained from all participants prior to assessment.

### Salivary sample collection

**Figure 1:**
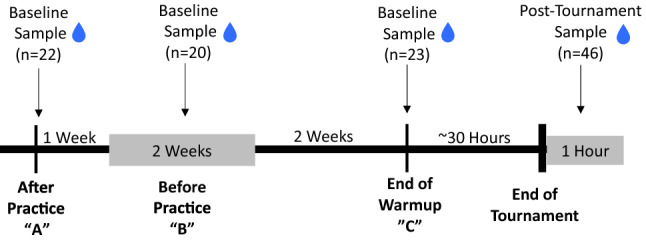
Diagram of sampling conditions relative to post-tournament samples. Salivary samples from 22 athletes were collected after a practice (“A”) held 5 weeks prior to the tournament. Salivary samples from 20 athletes were collected before a practice (“B”) held 2–4 weeks before the tournament. Salivary samples from 23 athletes were collected after warm-up on Day 1 of a two-day tournament (“C”). Post-tournament salivary samples were collected from 46 athletes within an hour.

Baseline saliva samples were collected in one of three conditions based on team availability: (i) approximately 1 h after practice; (ii) after a swimming ‘warm-up’ and prior to the first tournament game; (iii) or before practice (≥ 22 h after the last practice). The distribution of these conditions across teams are reported in Table 1. This means that the time between pre-tournament and post-tournament samples was (i) 5 weeks, (ii) approximately 30 h, or (iii) 2–4 weeks, respectively (Fig. 1). Post-tournament samples were collected within 1 h of the end of the final tournament game, meaning that the biomarkers were sampled anywhere from 10 min to 32 h after the last validated head impact in a 2-day tournament or 10 min to 7 h after the last validated head impact in the 1-day tournament (Women’s Varsity).

**Table 1:** Athlete demographic information.

	Men		Women	
	Club n = 14	Varsity n = 11	Club n = 18	Varsity n = 22
Age (years ± SD)	19.8 ± 2.1	20.4 ± 1.3	19.9 ± 2.0	19.6 ± 1.2
**Race/ethnicity (n)**				
White	8	11	6	12
Hispanic/Latino	4	0	8	5
Asian	1	0	3	0
Hawaiian/Pacific Islander	1	0	1	0
Not Reported	0	0	0	5
Lifetime years playing water polo (years ± SD)	5.8 ± 2.4	9.8 ± 3.3	5.8 ± 3.4	9.5 ± 3.1
Months playing water polo in prior year (months ± SD)	7.0 ± 3.9	11.7 ± .6	6.9 ± 4.7	10.6 ± 1.2
Average time spent playing in prior year (hours/week ± SD)	9.5 ± 3.1	21.3 ± 3.6	9.0 ± 2.6	16.3 ± 9.0
Number of players reporting prior mTBI (n/%)	2 (14%)	2 (19%)	4 (22%)	3(14%)

Ultimately, baseline samples were collected from 31 Division I athletes and 32 Club athletes, using the passive drool method according to previously established protocols^48^. Approximately two ml of unstimulated whole saliva was obtained from each participant. Samples were immediately frozen at − 20 °C at the time of collection, then stored at − 80 °C. At one tournament (Men’s Varsity), an appropriate cooler was not available, and samples collected before and after the tournament were kept at 0 °C until they were transferred to a − 80 °C freezer approximately 24 h later.

### Salivary sample processing

At the time of use, saliva samples were thawed and centrifuged at 10,000*g* for 10 min at 4 °C to remove insoluble material and cellular debris. Supernatants were collected and used for all assays. S100B levels were quantified in saliva samples using a commercially available ELISA kit (Millipore, EZHS100B-33K SDS) according to the manufacturer’s protocol using 25 μl of saliva supernatant per well, but with a 2-h incubation period for the test samples with the coating antibody. Samples were diluted 1:1 with the Assay Buffer containing lyophilized Matrix Solution. The recombinant S100B protein standard provided with the kit was used for generating the standard curve. Recovery of a known amount of S100B in the saliva matrix was established by analyzing n = 6 saliva samples from normal individuals at baseline and then spiked in with the S100B standard at 187 pg/ml. The recovery of S100B in the saliva matrix was 99.7% ± 20.01%. Each measurement was performed in duplicate by operators blinded to the clinical state of the participant. The lower limit of detection (LOD; lowest standard reading significantly above blank) was calculated as the blank signal plus two standard deviations from the mean and equaled 1.56 pg/ml. No S100B samples fell below this threshold. Test samples with a coefficient of variation (CV) of > 20% were excluded from analysis. The antibody pair used in this assay measures Human S100B and has no cross-reactivity with S100A1, S100A6 and S100A13.

NfL levels in saliva samples were measured using the Neurofilament light R-PLEX Antibody Set (Mesoscale Discovery) essentially according to the manufacturer’s protocol using 25 μl of saliva supernatant per well and using a 2-h incubation period for the test samples with the coating antibody. Saliva samples were diluted 1:4 with the MSD Diluent 12. The human NfL calibrator provided with the kit was used for generating the standard curve. Recovery of a known amount of NfL in the saliva matrix was established by analyzing n = 6 saliva samples from normal individuals at baseline and then spiked in with the NfL calibrator at 200 pg/ml. The recovery of NfL in the saliva matrix was 118% ± 9.54%. Each measurement was performed in duplicate by operators blinded to the clinical state of the participant. The lower limit of detection (LOD; lowest standard reading significantly above blank) was calculated as the blank signal plus two standard deviations from the mean and equaled 1.01 pg/ml. Samples from 18 of 64 athletes fell below this threshold at both pre- and post-tournament collections. Samples from 11 of 64 athletes fell below this threshold pre-tournament but not post-tournament. Samples from 4 of 64 athletes fell below this threshold post-tournament but not pre-tournament. LOD value was assigned to samples that were below the detection limit. Samples with CV > 20% were excluded from analysis.

### Head impact monitoring

Participants were fitted with Smart Impact Monitor (SIM-G) sensors that relayed impact data to a sideline device (Triax Technologies; Norwalk, CT). Each SIM-G sensor was inserted into an athlete’s water polo cap that had been modified to include a Velcro pocket designed to couple the sensor with the wearer’s occipital protuberance. Laboratory evaluations of the SIM-G demonstrate that it can record peak kinematic values of head impacts when coupled tightly to the occipital protuberance in a headband, and that the SIM-G performs comparably when secured using a water polo cap^49^.

Head impacts were monitored for the men’s and women’s club teams and men’s varsity team during a two-day tournament consisting of four games against opponents of respective sex and level of play. Data for four men’s club athletes were collected at a separate two-day tournament consisting of three games. Women’s varsity team athletes were monitored during a single-day tournament in which they played four games against other women’s varsity teams. The SIM-G sensors recorded the peak linear acceleration (PLA), peak rotational acceleration (PRA), and peak rotational velocity (PRV) associated with each head impact.

Impacts registering a PLA < 16 g were filtered automatically (i.e., not recorded) by the sensors’ standard recording threshold. The SIM-G sensors’ non-impact transient filter, based on algorithms that have demonstrated poor reliability^49^, was disregarded. Instead, to verify the validity of recorded accelerative events as head impacts, seven research assistant staff members performed visual inspection of two angles of video recordings that were time-synced with impact data for each game. These visual inspections were then integrated methodically as detailed below.

After training on proper coding of video by senior project staff, each reviewer independently reviewed impacts. Due to limitations in head impact sensor technology, reviewers were instructed to confirm accelerative events as ‘true’ head impacts if the following conditions were met: (i) the mechanism and location of an impact (on the head) was visible on video; (ii) the athlete’s capped head was entirely above the water; and (iii) the cap remained coupled to the athlete’s head during impact. Accelerative events that were deemed as ‘true’ head impacts by a majority of the reviewers (i.e., four or more) were included in subsequent analyses and those that were deemed as ‘true’ head impacts by only one or none of the reviewers were rejected as false positives. These accelerative events were discarded from any further analysis. Accelerative events that only two or only three reviewers deemed as ‘true’ head impacts were included in the analyses if it was determined that inclusion criteria could be met after further review by senior project staff; otherwise, these data were also rejected as false positives and excluded from further analysis. Interrater agreement was high (96.9%) and interrater reliability, which considers ‘chance’ agreement, was also high (Fleiss’ Kappa = 0.740)^50^, considering that seven independent raters used binary ratings.

To account for the known inaccuracies of the kinematic measures (PLA, PRA, PRV) recorded by the SIM-G^49^, the kinematic measures of all impacts confirmed through this method were subjected to a Principal Component Analysis (PCA) to produce a new composite measure of relative impact severity in a procedure described by Greenwald et al.^51^. Weighted cumulative head impact exposure (wCHI) was computed as the sum of all principal component scores, as a representation of the frequency and cumulative impact magnitude sustained by each athlete during the tournament.

### Statistical analysis

Biomarker concentrations are not expected to be normally distributed, and thus assumptions about residual distributions underlying parametric tests are likely to be violated. Thus, to test our primary hypothesis, four separate gamma generalized linear models (GzLMs) with a log link function were fit using the *GENLIN* function (SPSS 25; IBM Corporation) with post-tournament biomarker concentrations (post-S100B, post-NfL) as the response variable, head impact exposure (Frequency, wCHI) as predictors, and baseline biomarker concentrations as covariates (base-S100B, base-NfL). Fitting the error term with a Gamma distribution is common for data that are positive (greater than zero) and have positive skewness (i.e., a long tail on the distribution away from zero). The log link function enables coefficients to be easily transformed into the original units, making this approach preferable to an ordinary least squares regression analysis of log transformed variables. Spearman correlation coefficients confirmed that the predictor variables (baseline biomarker concentrations and head impacts) were not correlated.

Our group previously demonstrated that exposure differed between males and females competing at the club level^46^, but the potentially interactive effect of competition level is unknown in water polo. Thus, we characterized head impact exposure for each team and tested for differences between teams based on sex and level of competition. A gamma GzLM with a log link function was used to test for differences in wCHI, as the response variable, and group (sex, competitive level) as the predictor variables. For a model predicting impact frequencies, the error term was fit with a Poisson distribution with a log link function, an approach commonly used for ‘count’ data.

An unintended outcome of our baseline data collection procedures was that baseline sampling conditions differed between participants in a systematic way, based on time since the leaving the pool (i.e., for a practice or warm-up). Given that physical exertion may confound the interpretation of biomarkers of brain injury, we also tested a post hoc hypothesis that baseline biomarker concentrations (response variable) differed between the pre-practice condition (≥ 22 h post-exertion) and the post-practice and post-warmup conditions using a gamma GzLM with a log link function.

Unless otherwise specified, all models exhibited sufficient fit based on an ‘omnibus’ likelihood ratio Chi-square test (p < 0.001) of the null hypothesis that the fitted model was not different than the intercept-only model (i.e., a test that the model was able to predict the response variable better than would be expected by chance)^52^. Goodness of fit was further assessed by visual inspection of diagnostic plots of deviance residuals against predicted responses to confirm the absence of any trend. Deviance computed from GzLMs represents lack of fit relative to a saturated model (i.e., a model for which a separate parameter is estimated for each participant) and is comparable to the residual sum of squares in ordinary least squares regression. Thus, deviance residuals represent the degree to which each participant contributed to the overall model deviance. The deviance-predicted relationship was confirmed to be trend free, and a deviance test statistic was also computed as the difference between the log-likelihood of the model-of-interest and the log-likelihood of the saturated model^53^. We tested the null hypothesis that the saturated model did not exhibit a better fit than the model-of-interest by comparing the deviance test statistic to the Chi-square (χ^2^) distribution. Unless otherwise specified, we failed to reject the null hypothesis for (p > 0.05), meaning that the saturated model was not a better fit than the model-of-interest.

For each model, inferential tests were performed by Type III Wald tests (χ^2^) of the null hypothesis that a parameter estimate was equivalent to zero after adjusting for the influence of the other predictors in the model. Specifically, the Wald statistic is calculated as the squared ratio of the coefficient (B) to its standard error. This means that given α = 0.05 (a Type I error rate of 5%) and β = 0.2 (a Type II error rate of 20%), we have an 80% probability (power) to detect response–predictor relationships ≥ 1.95 times larger than the modeled standard error. Rate ratios (RR) are reported as the exponential of point estimates (B) for each coefficient and can be interpreted to indicate a factor (multiplicative) increase in the response variable for a one unit change in the predictor variable, while controlling for other factors or covariates in the model.

### Power analysis by simulation

Participants were recruited from the population of all competing University water polo players, without an a priori power analysis. Whereas sensitivity analyses can be performed for parametric tests based on sample size, preferred Type I/II error rates, and the degrees of freedom of that test, the complexity of GzLMs makes these approaches impractical^54^. Therefore, statistical power, which is defined as the probability of correctly rejecting the null hypothesis, was formally estimated via a four-step resampling procedure in Matlab 2020b (MathWorks, Natick, MA) for each model testing our primary and secondary hypotheses. First, for each model fitted to predict post-tournament salivary biomarker, a simulated response variable was constructed based on published findings relating head impacts and serum S100B and NfL in experimental studies of soccer ball-to-head impacts. We focused specifically on these studies for three reasons: (i) those impacts have exhibited similar kinematics to impacts reported here, (ii) head impact frequency is carefully controlled in a laboratory setting, and (iii) biomarker sampling 24 h post-exposure is comparable to the timing of our post-tournament sampling relative to impacts that occurred on day 1 of the tournament. Post-tournament salivary NfL was simulated as an increase beyond baseline NfL of 0.066 pg/ml per head impact with a random additive error term of ± 0.022 pg/ml per head impact^36^. Post-tournament salivary S100B was simulated as an increase from baseline S100B of 1.95 pg/ml per head impact with a random additive error term of ± 1.6 pg/ml per head impact^55^.

Second, identical models were fit to the simulated biomarker variable to test for group and individual effects as described above. Third, for each model an iterative process was performed (n = 1000) in which a random response variable was generated from the model fitted to the simulated data (*random* function), that simulation-fitted model was then refit using the new responses (*refit* function), and the p-values from the resulting Wald test and omnibus likelihood ratio test were recorded. Fourth, the percentage of refitted models rejecting the null hypothesis for each test (p < 0.05) was calculated to represent the estimated statistical power of that model. With respect to the Wald tests of the null hypothesis that modeled coefficients were non-zero, we report that tests of a relationship between head impact frequency and post-tournament S100B and NfL were powered at 54.5% and > 99.9% respectively. Tests of a relationship between the cumulative magnitude-weighted exposure and post-tournament S100B and NfL were powered at 55.9% and > 99.9% respectively. The low power (< 80%) for detecting changes in S100B is discussed later in this report. With respect to omnibus likelihood ratio tests, all models were powered at > 99.9% to reject the null hypothesis that the model-of-interest exhibited a fit that was not better than the intercept-only model.

## Results

### Athlete characteristics

Demographic information, lifetime and 12-month experience in water polo are reported in Table 1. Biomarker medians and interquartile ranges (IQR) are organized by team and sampling condition in Table 2. No participants reported using any supplements, nor did any participants report competing in any other sports during the 2018–2019 season. No participants reported sustaining a mTBI during the tournament at which they were monitored.

**Table 2:** Baseline biomarker samples by condition and team.

Timing of baseline collection	Men		Women	
	Club n = 14	Varsity n = 11	Club n = 18	Varsity n = 22
**Before practice (n/%)**	4 (29%)	0	16 (89%)	0
S100B, pg/ml (median/IQR)	58.51 (22.95, 335.70)	–	38.36 (17.77, 52.27)	–
NfL, pg/ml (median/IQR)	3.39 (1.12, 25.38)	–	2.39 (1.03, 29.5)	–
Players with post-tournament samples (n/% of baseline)	2 (50%)		11 (69%)	
**~ 1 h after practice (n/%)**	0	0	0	22 (100%)
S100B, pg/ml (median/IQR)	–	–	–	37.35 (14.46, 65.76)
NfL, pg/ml (median/IQR)	–	–	–	1.03 (1.03, 6.91)
Players with post-tournament samples (n/% of baseline)	0	0	0	15 (68%)
**After warm-up (n/%)**	10 (71%)	11 (100%)	2 (11%)	0
S100B, pg/ml (median/IQR)	51.26 (23.85, 105.72)	156.84 (41.38, 235.38)	49.04 (18.01)	–
NfL, pg/ml (median/IQR)	1.01*	4.93 (1.97, 23.89)	1.98 (1.01)	–
Players with post-tournament samples (n/% of baseline)	9 (90%)	8 (73%)	1 (50%)	0

*Indicates all samples were the same and thus median, 1st quartile, and 3rd quartile are equal.

### Group differences in head impacts

**Figure 2:**
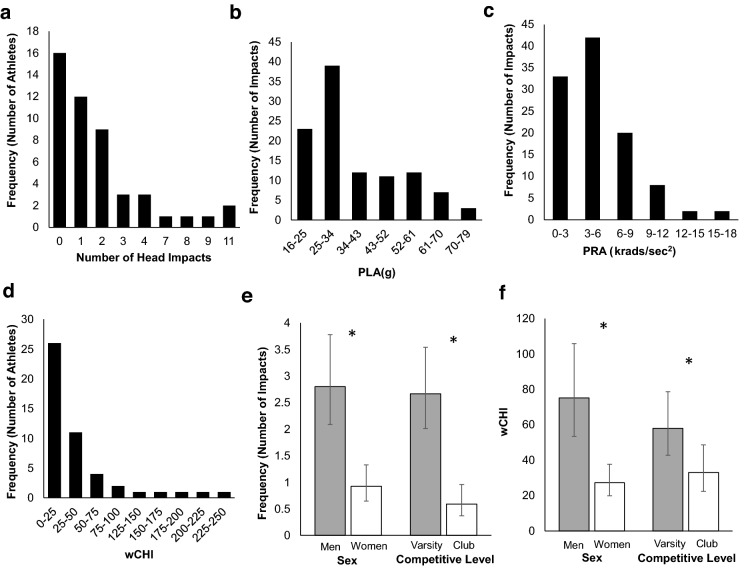
Differences in head impact exposure by competitive team. (**a**) Distribution of head impact frequency per athlete. (**b**) Distribution of peak linear acceleration (PLA) per impact. (**c**) Distribution of peak rotational acceleration (PRA) per impact. (**d**) Distribution of cumulative head impact magnitude (wCHI) per athlete. (**e**) Predicted (95% confidence intervals) number of head impacts sustained by men’s (n = 25), women’s (n = 40), club (n = 32), and varsity (n = 33) teams. (**f**) Predicted (95% confidence intervals) wCHI sustained by men’s (n = 25), women’s (n = 40), club (n = 32), and varsity (n = 33) teams. *Denotes significant difference between groups, p < 0.05.

The SIM-G sensors recorded 1403 accelerative events across all monitored water polo games. A total of 107 accelerative events were verified as head impacts after video review, with each athlete sustaining between 0 and 11 head impacts during tournament competition (median: 1 head impact) (Fig. 2a). These impacts had a median PRA of 3.8 krads/s^2^ (IQR 2.45–6.45 krads/s^2^) (Fig. 2b) and median PLA of 31.91 g (IQR 25.9–46.19 g) (Fig. 2c). The median wCHI sustained across the tournament per athlete was 17.76 (IQR 0–42.48) (Fig. 2d). Men sustained more impacts than women [RR = 3.515; 95% CI 2.358, 5.244; χ^2^ = 38.030; p < 0.001] and varsity athletes sustained more impacts than club athletes [RR = 5.130; 95% CI 3.121, 8.440; χ^2^ = 41.548; p < 0.001] (Fig. 2e). Men sustained greater wCHI than women [RR = 2.959; 95% CI 2.018, 4.341; χ^2^ = 30.530; p < 0.001] and varsity athletes sustained greater wCHI than club athletes [RR = 1.976; 95% CI 1.370, 2.846; χ^2^ = 10.421; p = 0.001] (Fig. 2f).

### Dose–response between salivary biomarkers and head impact exposure

**Figure 3:**
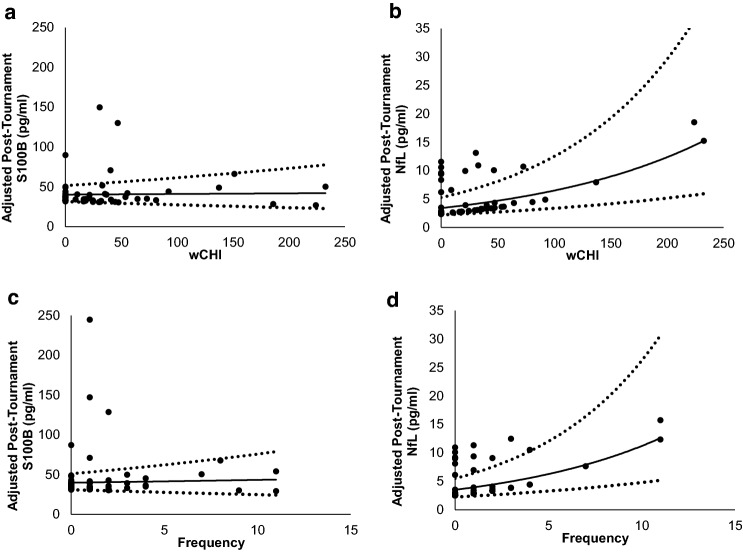
Dose–Response relationships between head impact exposure and salivary biomarkers. (**a**) Modeled relationship between post-tournament salivary S100B and the number of head impacts sustained during the tournament after adjustment for pre-tournament S100B (n.s.). (**b**) Modeled relationship between post-tournament salivary S100B and wCHI sustained during the tournament after adjustment for pre-tournament S100B (n.s.). (**c**) Modeled relationship between post-tournament salivary NfL and the number of head impacts sustained during the tournament after adjustment for pre-tournament NfL (RR = 1.151; 95% CI 1.031, 1.285; p = 0.025). (**d**) Modeled relationship between post-tournament salivary NfL and wCHI sustained during the tournament after adjustment for pre-tournament NfL (RR = 1.008; 95% CI 1.002, 1.013; p = 0.014). Dashed lines represent 95% Confidence intervals.

Saliva was sampled between 5 min and 28.8 h after the final recorded head impact (median = 20.8 h; IQR 6.0–26.0 h). When controlling for baseline S100B, post-tournament S100B was not associated with head impact exposure [RR < 1.001; p > 0.483] (Fig. 3a,b). When controlling for baseline NfL, post-tournament NfL was positively associated with the number of head impacts sustained during the tournament [RR = 1.151; 95% CI 1.031, 1.285; χ^2^(1) = 5.050; p = 0.025] and wCHI incurred by those impacts [RR = 1.008; 95% CI 1.002, 1.013; χ^2^(1) = 6.086; p = 0.014] (Fig. 3c,d). This means that post-tournament salivary NfL increased by a factor of 1.151 for every head impact and a factor of 1.008 for every unit of wCHI sustained by an athlete during the tournament. Individual and group changes in salivary biomarker concentrations from pre- to post-tournament are depicted in Supplemental Figs. S1 and S2 respectively.

### Biomarker differences between baseline sampling conditions

**Figure 4:**
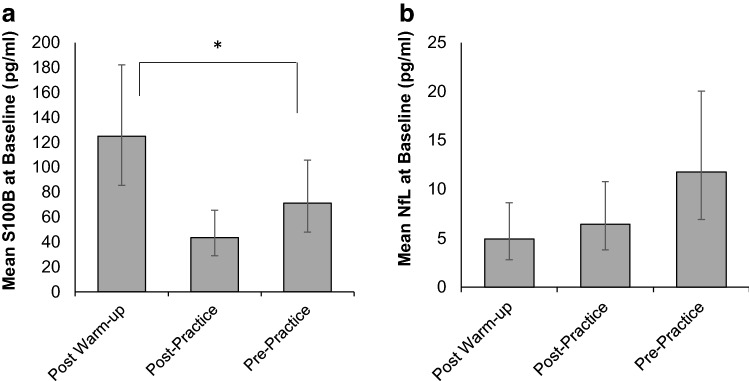
Differences in baseline salivary biomarker concentrations by sampling condition. Modeled relationship between baseline salivary S100B (**a**) and NfL (**b**) and sampling condition ordered by increasing proximity to physical exertion: ~ 15 min post-warmup, ~ 1 h after practice, and before practice (≥ 22 h after the last practice). *Denotes significant difference between conditions, p < 0.05.

There was an effect of baseline sampling condition on baseline S100B such that concentrations were higher in samples collected after warm-up compared to samples collected before practice [RR = 1.754; 95% CI 1.013, 3.037; χ^2^(1) = 4.028; p = 0.045] (Fig. 4). Baseline sampling condition was not a sufficient predictor of baseline NfL [χ^2^(2) = 5.203; p = 0.074].

## Discussion

We examined associations between exposure to repetitive head impacts during a water polo tournament and changes in salivary expression of S100B and NfL, candidate protein biomarkers of astrogliosis and axonal damage in mTBI. Our primary novel finding is a dose–response relationship between the frequency and cumulative magnitude (wCHI) of head impacts sustained during a water polo tournament and changes in salivary NfL expression.

Most of the impacts we observed had a peak linear acceleration component < 32 g and a peak rotational acceleration component < 4 krads/s^2^, making them comparable to impacts measured in soccer and lacrosse with the same sensor used in the current study^56,57^. Note that the poor false-positive rate of the SIM-G in this study (92.4%) is well documented in water polo^45,46^, and is consistent with limitations of similar head impact sensors in land-based sports^58^, underscoring the importance of video review for interpretation of data collected with these sensors^59^. Likewise, it is quite unlikely that the sensors have a significant false-negative rate and, when considered together with the high interrater reliability between independent observers, it is also unlikely that exposure, as measured by the frequency of head impacts, is underestimated in our sample. Despite the SIM-G’s potentially inaccurate kinematic measurements^49^, head-to-ball impacts studied in a laboratory setting produced similar head kinematics to those reported here and were associated with increases in serum NfL^36^. These kinematic parameters may be meaningful for understanding mechanical strain placed on brain tissue, which appears to be correlated with changes in blood–brain barrier integrity from pre- to post-competition in mixed martial arts athletes^14^. Therefore, enhanced post-tournament salivary NfL expression in this sample suggests that mechanical loading sustained during a single tournament is capable of causing both axonal damage and blood–brain barrier disruption. To our knowledge, this is the first report of changes in putative salivary protein biomarkers of brain injury in asymptomatic athletes, and these findings contribute to a growing body of literature supporting the utility of NfL for monitoring the physiological effects of repeated mechanical loading of the head.

Relative to other biomarkers of brain injury, NfL is believed to be longer lasting, with an in vivo half-life in rodent models of 3–6 weeks^60^. Human studies have reported elevated NfL in serum for months^31^ and years after injury^61^. On one hand, NfL may be useful for monitoring physiological recovery after sport-related concussion, which is now widely considered to outlast symptomatic recovery^62^. Although recovery is often monitored at-home, return-to-play protocols are typically executed by athletic trainers, sports medicine physicians, or physical therapists in a variety of out-patient settings—not all medical facilities—where taking regular blood samples may be impractical or unsafe, particularly for a recovering athlete. The convenience and safety of sampling saliva makes this a promising application worthy of future exploration. On the other hand, a long NfL half-life may also confound interpretation of post-tournament NfL when controlling for baseline NfL that was sampled many weeks earlier. In other words, it is possible that increased NfL in those participants might be due to repetitive head impacts sustained during unmonitored practices between the baseline sample and the tournament rather than at the tournament.

Our data do not support a relationship between changes in S100B and head impact exposure, adding to an already ambiguous body of literature concerning the utility of S100B as an indicator of head mechanical loading. It is from this literature that our power analysis simulation revealed that our models may not be sufficiently powered to detect potential group-wise differences or dose–response changes in S100B. It cannot be ruled out that the lack of a dose–response relationship merely represents a Type II error. However, beyond potential sample size limitations, there are also plausible physiological explanations. Compared to NfL, S100B turnover is relatively rapid^63^, meaning that the lack of an association between post-tournament S100B and head impact exposure could be explained by a long delay between the last head impact and saliva sampling. Greater S100B was observed at baseline in men compared to women, but reports of sex differences in serum S100B have been mixed, without any biologically plausible explanation^64,65^. In the current study it is possible that differences in baseline sampling conditions could have contributed to this pattern: Baseline samples from men were mostly collected immediately after a pre-tournament warm-up, which in water polo typically consists of a short bout of intense swimming and skill work, whereas baseline samples from women were mostly collected before or (approximately 1 h) after a standard practice.

This pattern is consistent with a widely accepted theory that serum S100B expression could be enhanced following physical exertion, which confounds the interpretation of circulating S100B as a biomarker of sport-related brain injury^66,67^. During periods of stress and catabolism S100B on skeletal and cardiac myocytes plays a probable role in calcium signaling^68^, while S100B is secreted by adipocytes as an adipokine, possibly to orchestrate inflammatory cascades or to stimulate glycolysis^69,70^. This could explain why Dietrich et al. observed increases in serum S100B in 14 of 16 participant after a single open-water swimming race and why, in the current study, men exhibited greater salivary S100B concentrations at baseline than women^71^. On the other hand, the women’s varsity team played four games in one day, meaning that their exertion was more condensed than in athletes from the other three teams, who competed in two-day tournaments. In the context of our primary hypothesis, this means that some of the athletes expected to have the lowest baseline S100B concentrations, on account of sampling conditions that were distal to exertion, would also be expected to exhibit the greatest exertion-related increases in salivary S100B post-tournament, therefore confounding our attempt to predict S100B changes from measures of head mechanical loading.

Ultimately, no data were collected in the current study that could fully resolve a potential confounding effect of physical exertion on pre- to post-tournament changes in biomarker concentrations. Playing time or other measures of exertional load (e.g., heart rate) may be helpful for teasing out these confounding effects and clarifying the source of salivary S100B in future studies^27^. We based our power simulations on the findings of Huibregtse et al., who likely used a kicking-control group as a means of controlling for this exertional confound^55^. However, the only 24-h effect those authored reported was elevated S100B within the soccer-heading group, not an interaction effect. In other words, the variable effects they reported may have been confounded by exertion, thereby contributing to the low power estimates we computed for S100B models in the current study. Also note that we observed much smaller effects of head impact exposure on changes in S100B then that group did. Ultimately, we do not consider the null findings we report to represent Type II errors (i.e., that the small effects we observed are real and we did not have a large enough sample size to reject the null hypothesis), and consider it more likely that unmeasured factors, like exertion, contributed to ‘error’ in these models. It is also worth noting that the women’s varsity team played four games in one day, meaning that exposure and exertion may have been more condensed for some of those athletes compared to athletes from the other three teams competing in two-day tournaments. On the other hand, it is also possible that the lack of a relationship between head impact exposure and S100B could be due to the lesser movement-associated head vibrations experienced by water polo players relative to athletes engaged in land-based sports (i.e., regular head accelerations/decelerations from running) that have been suggested to enhance serum S100B^66^, independent of exertion^72^.

One recent report found salivary and serum S100B concentrations to be strongly correlated, demonstrating comparable performance when used to distinguish adults with suspected mTBI from controls^41^. Likewise, due to its relatively low molecular weight, we expect that levels of salivary NfL would be similarly related to those in blood, even though we did not directly compare saliva and blood levels of NfL. Constituents from the blood can enter the saliva via transcellular transport, passive intracellular diffusion or active transport, and thus, salivary levels of these markers could reflect the overall circulating levels in the body^73^. On the other hand, salivary analytes that can discriminate between brain injured and healthy populations, or as in the present study, be predicted by a continuous measure of mechanical loading of the head, may be clinically important whether or not serum–saliva associations are weak or altogether unknown^74^. These possibilities warrant the study of dynamic serum–saliva associations and consideration for a moderating role of head mechanical loading in these relationships. Even then, the exact mechanisms through which NfL is transported to the blood from the central nervous system remain unknown. Given that even mild head injuries can result in a compromised blood–brain barrier, the changes in NfL levels observed could reflect leakage from the brain^14^, but it is just as possible that circulating concentrations are independent of blood–brain barrier disruption^75^. This is not trivial, as the interpretation of serum or salivary biomarkers to represent brain injury would be confounded by inter- and intra-individual differences in rates of release. Future studies comparing advanced neuroimaging of the blood–brain barrier with changes in salivary S100B and NfL will shed light on this issue, perhaps providing stronger support for these biomarkers than serum–saliva associations alone.

The present findings are also consistent with our previous report that changes in brain slow-wave synchrony over one season in water polo players is directly and linearly associated with the magnitude and frequency of head impacts (wCHI) and with frequency taken alone, i.e., without consideration of impact magnitude^47^. ‘Hyperconnectivity’ is theorized as a fundamental, compensatory response of brain functional networks in the face of microstructural perturbation^76^. It is possible that increased functional connectivity over a season of water polo competition may be attributed to axonal damage from repetitive head impact exposure, as inferred from the dose–response between impact exposure and NfL observed in the current study. One structural MRI study of 21 female former soccer players reported progressive widening of the sulci and low-intensity punctate regions at the grey-white matter interface, patterns suggestive of a “water hammer” injury and, the authors speculate, early signs of neurodegeneration^77^. The authors of that study hypothesize that their imaging findings may have been explained by chronic release of NfL through a compromised blood brain barrier that was caused by repeated sport-related head impacts. The patterns we report here lend support to their hypotheses by directly relating head impact exposure with acute increases in circulating NfL, neither of which were measured in that study. Collectively, these findings suggest that salivary NfL may one day be used to monitor brain health in collegiate athletes, during and beyond their competitive careers.

In conclusion, our findings demonstrate that S100B and NfL are detectable in saliva, a biofluid that is simpler and safer to collect than blood, and that there is a dose–response relationship between changes in salivary NfL and head impacts sustained during water polo competition. Our data also encourage careful selection of baseline sampling, particularly for biomarkers, like S100B, which have demonstrated sensitivity to exertion. Future research is needed to relate these salivary biomarkers to validated measures of brain injury in clinical populations.

## Supplementary Information

Supplementary Figures.
