# Supplementary material for: Salivary S100 calcium-binding protein beta (S100B) and neurofilament light (NfL) after acute exposure to repeated head impacts in collegiate water polo players
Source: Sci Rep. 2022 Mar 2;12:3439. doi: 10.1038/s41598-022-07241-0 (PMC8891257; doi:10.1038/s41598-022-07241-0)
Supplement: Supplementary file 1 — Supplementary Figures. [file 41598_2022_7241_MOESM1_ESM.pdf]

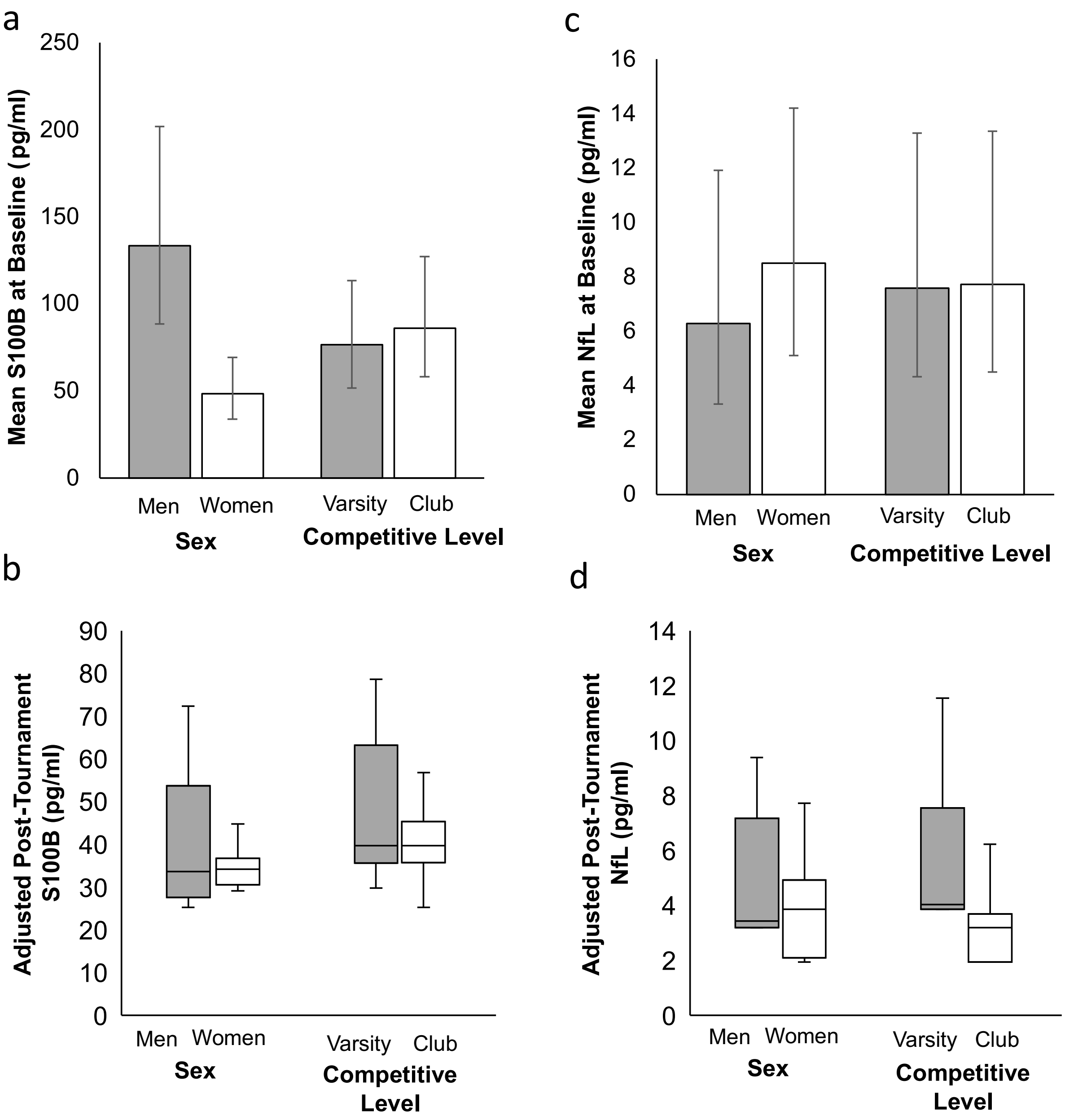

Figure S1: Modeled salivary biomarker concentrations by team. a) Predicted (95% confidence intervals) baseline salivary S100 calcium-binding protein beta (S100B) measured from men's (n = 24), women's (n = 37), club (n=32), and varsity (n=29) teams. b) Median, interquartile range (box), and extremes (whiskers) of predicted post-tournament salivary S100B measured from men's (n = 21), women's (n = 24), club (n=23), and varsity (n=22) teams adjusted for baseline

S100B. c) Predicted (95% confidence intervals) baseline salivary neurofilament light (NfL) from men's (n = 20), women's (n = 39), club (n=31), and varsity (n=28) teams. d) Median, interquartile range (box), and extremes (whiskers) of predicted post-tournament salivary NfL measured from men's (n = 18), women's (n = 26), club (n=23), and varsity (n=21) teams adjusted for baseline NfL. *Denotes significant difference between groups, $p < .05$

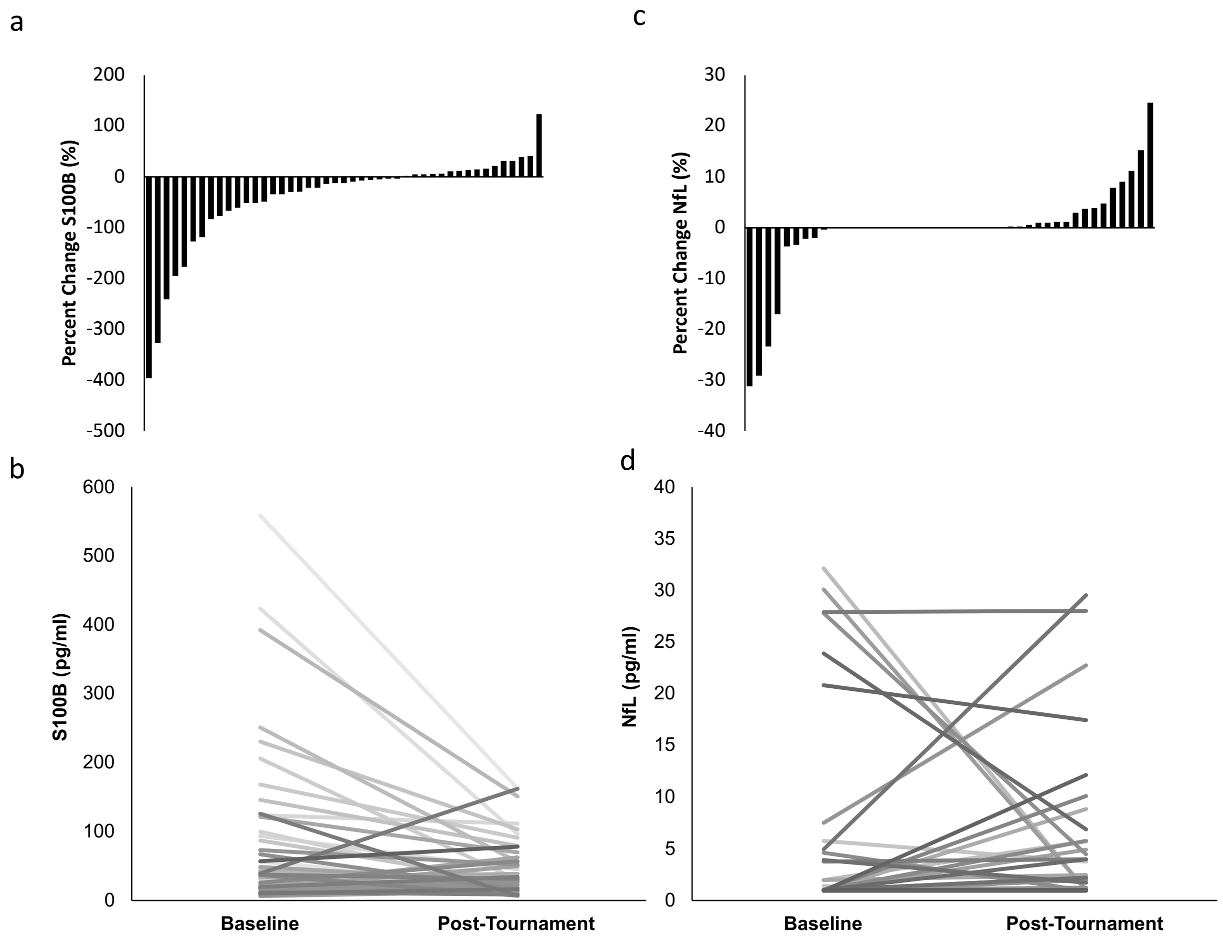

Figure S2: Individual changes in salivary biomarkers. % change in salivary S100B (a) and NfL (C) from baseline to post-tournament. Individual changes in S100B (b) and NfL (d) from baseline to post-tournament.
